# Variability Identification and Uncertainty Evolution Characteristic Analysis of Hydrological Variables in Anhui Province, China

**DOI:** 10.3390/e27030305

**Published:** 2025-03-14

**Authors:** Xia Bai, Jinhuang Yu, Yule Li, Juliang Jin, Chengguo Wu, Rongxing Zhou

**Affiliations:** 1 College of Civil Engineering, Anhui Jianzhu University, Hefei 230601, China; baixia0516@163.com (X.B.); 500516@ahjzu.edu.cn (J.Y.); wule9896@163.com (Y.L.); 2 College of Civil Engineering, Hefei University of Technology, Hefei 230009, China; jinjl66@126.com; 3 Institute of Water Resources and Environmental Systems Engineering, Hefei University of Technology, Hefei 230009, China; 4 School of Environment and Energy Engineering, Anhui Jianzhu University, Hefei 230601, China; zhourx11@163.com

**Keywords:** variability identification, uncertainty, entropy, LTR, CM, Anhui Province

## Abstract

Variability identification and uncertainty characteristic analysis, under the impacts of climate change and human activities, is beneficial for accurately predicting the future evolution trend of hydrological variables. In this study, based on the evolution trend and characteristic analyses of historical precipitation and temperature sequences from monthly, annual, and interannual scales through the Linear Tendency Rate (LTR) index, as well as its variability point identification using the M–K trend test method, we further utilized three cloud characteristic parameters comprising the average *Ex*, entropy *En*, and hyper-entropy *He* of the Cloud Model (CM) method to quantitatively reveal the uncertainty features corresponding to the diverse cloud distribution of precipitation and temperature sample scatters. And then, through an application analysis of the proposed research framework in Anhui Province, China, the following can be summarized from the application results: (1) The annual precipitation of Anhui Province presented a remarkable decreasing trend from south to north and an annual increasing trend from 1960 to 2020, especially in the southern area, with the LTR index equaling 55.87 mm/10a, and the annual average temperature of the entire provincial area also presented an obvious increasing trend from 1960 to 2020, with LTR equaling about 0.226 °C/10a. (2) The uncertainty characteristic of the precipitation series was evidently intensified after the variability points in 2013 and 2014 in the southern and provincial areas, respectively, according to the derived values of entropy *En* and hyper-entropy *He*, which are basically to the contrary for the historical annual average temperature series in southern Anhui Province. (3) The obtained result was basically consistent with the practical statistics of historical hydrological and disaster data, indicating that the proposed research methodologies can be further applied in related variability diagnosis analyses of non-stationary hydrological variables.

## 1. Introduction

People understand hydrological rules based on long-term observational data with a consistent hypothesis. However, persistent human activities and climate change have altered the natural patterns of hydrological elements, resulting in hydrological variability [1,2]. This will lead to deviations in hydrological and water resources analyses, errors in decision making regarding water resource management, and even the design or construction of water conservancy projects [2,3]. Meanwhile, regional hydrological variables will also present spatio-temporal variation and even abrupt changing trends under the influences of the global changing environment, which will also lead to the occurrence of extreme disaster events comprising floods, heat, droughts, etc. [4,5]. A critical frontier is how to extract natural hydrological patterns from variability-influenced elements, and then accurately characterize water resource features so as to ensure water security and sustainable socioeconomic development. Therefore, the variability identification of hydrological variables and quantitative analysis of their uncertainty characteristic is essential to water resource management, climate prediction, and disaster prevention, which are becoming the frontier and key topics of the hydrology research field [6,7].

The essence of the variability identification analysis of the hydrological series is to recognize the variability point based on the long-term evolution trend test, and the recognition results will be beneficial for conducting an inconsistency analysis of hydrologic variable series. Firstly, the commonly applied approaches for the variability identification analysis of hydrological variables include time series analysis, the cumulative anomaly method, the T and F test methods, the non-parametric Pettitt test, the Mann–Kendall test, the Wavelet analysis method, etc. [8,9,10,11]. For instance, Q. Zhang et al. (2011) applied the sliding T and U test methods to diagnose the variation features of the monthly mainstream runoff series of the Yellow River in China and indicated that the variability points downstream occur earlier than that of the middle and upper reaches [12]. A. J. Guo et al. (2014) and B. Kong et al. (2019) recognized the variability points of the mainstream runoff series of the Weihe River Basin and diverse staged flood seasons of the North Luo River Basin in China by means of the cumulative anomaly method, the Mann–Kendall test, and the ordered clustering approach [13,14]. And then, in terms of uncertainty analysis of the variation in hydrological variables, current research primarily focuses on quantifying the uncertainty features of a hydrological series by extracting its stochastic characteristic variables, including statistical parameters, distribution patterns, fitting parameters, etc., or by decomposing the non-consistent hydrological series into deterministic and stochastic components and then achieving a deviation in the uncertainty features of entire hydrological sequences via a frequency analysis of the stochastic component [15,16,17]. For instance, S. B. Song et al. (2012) suggested employing a conditional probability model to investigate the uncertainty features of hydrological sequences [18]. P. X. Gao (2019) proposed an innovative flood frequency calculation method considering the uncertainties of sample selection, fitting line type determination, and parameter estimation [19].

Based on the above, it can be demonstrated that system uncertainty mainly encompasses randomness, fuzziness, uncertainty, etc., and the inherent randomness and fuzziness of systems associated with human cognition predominantly determine the uncertainty variation direction of complex systems. Generally, randomness is generally expressed by the occurring probability of random events, and fuzziness arises from the ambiguity of system classification, which leads to the subjective uncertainty of human cognition and is mainly expressed by certainty or membership degrees. In this study, the Cloud Model (CM) method was utilized to depict the fuzzy uncertainty and dynamic variability features of a hydrological sequence before and after the variability point, which can provide a scientific basis for the uncertainty quantitative analysis of non-consistent hydrological variables.

Anhui Province, China, is located in the transition zone between the subtropical humid monsoon climate in the north and the warm semi-humid monsoon climate in the south. Due to the impacts of climate and topography, Anhui Province exhibits multiple transitional characteristics of climatically from south to north, topographically from land to ocean and geographically from high to low latitude. Y. Huang et al. (2012) pointed out that the precipitation is generally more abundant in the south compared to the north in Anhui Province [20]. B. Q. Tang et al. (2016) indicated that the occurrence frequency of drought and flood processes decreased evidently from south to north in Anhui Province, and the occurrence frequency of drought events in the western region is significantly higher than that in the eastern area of Anhui Province [21]. Y. T. Liu et al. (2017) pointed out that the climate parameters of Anhui Province exhibited a clear warming trend during the past 55 years, and abrupt variability point of annual average temperature occurred in 1996 [22]. Thus, it can be inferred that multiple hydrological variables of Anhui Province all present significant variability and even abruptly changing features, and the intention of this study is to further quantitatively discuss the variability features of historical precipitation (P) and temperature (T) sequences in diverse regions of Anhui Province by means of the Linear Tendency Rate (LTR), Mann–Kendall (M–K) test, and Cloud Model (CM) approaches. In detail, the structure of this manuscript is organized as follows: in Section 1, we provide the research background and the main findings related to the variability characteristic exploration of hydrological variables. Then, the study area, research framework, and primary methodologies utilized in this study are explicitly introduced in Section 2 and Section 3. In Section 4, we discuss the application results of the proposed research framework in Anhui Province, China, to verify its reliability in the field of the variability diagnosis analysis of hydrological variables. And finally, we summarize the primary findings and shortcomings of this study and also provide the research directions for the future.

## 2. Study Area and Research Framework

### 2.1. Study Area and Data Sources

**Figure 1:**
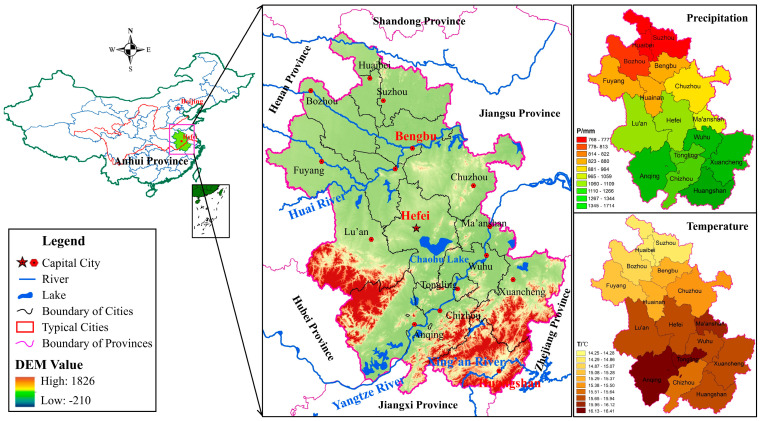
Geographical location of Anhui Province in China.

Anhui Province is located in the southeast of China, with a total land area of 140 thousand km^2^ and a permanent population of 61.21 million in 2023. The Huaihe and Yangtze Rivers flow across the Anhui Province, which divides the entire provincial area into northern, central, and southern regions. The average annual precipitation of Anhui Province is 800–1800 mm. Meanwhile, Anhui Province exhibits evident altitudinal variations with an average of approximately 300 m. The Huaibei Plain, characterized by its flat terrain at elevations of 20–50 m, is the lowest-altitude region in the province. In contrast, the west and south hilly and mountainous areas demonstrate higher elevations, ranging from 500 to 1500 m and 200 to 1800 m, respectively. As a major agricultural area, Anhui Province had a rapid population growth from 1960 to 1980, and the provincial population was about 50 million in 1980, which has reached 61.03 million in 2020. In addition, the provincial GDP reached CNY 3.87 trillion in 2020, and the provincial cultivated land area reached 5546 × 10^3^ hm^2^. As illustrated in Figure 1, the northern area of Anhui Province is mainly composed of plains, with average annual precipitation and temperature varying below 880 mm and 15.28 °C, and the central area has a terrain with dense river network and abundant water resources, with average annual precipitation and temperature varying between 880 and 1200 mm and 15.28 and 16.10 °C, while the average annual precipitation and temperature in the southern area vary above 1200 mm and 16.10 °C [23]. In addition, until the end of 2024, Anhui Province achieved a cultivated land area of 5.6 million hectares, displaying a consistent growth trend during the past four years. Currently, the provincial urbanization rate is 62%, with a targeted rate of over 73% by 2035. The annual average temperature in Anhui Province has shown a gradual rise in recent years. The period from 2022 to 2024 recorded three consecutive years as the hottest in recent history, with the average temperature in 2024 hitting 17.4 °C, i.e., the highest on record since 1961. Meanwhile, drought and flood disasters occur frequently in Anhui Province, which seriously threatens the food security and stable economic development of the entire province. Specifically, the occurrence frequency of flood and drought disasters in the northern area and Jianghuai hilly area is relatively higher, and the historical statistics show that the occurrence frequencies of major flood and severe drought disasters in the northern and Jianghuai hilly areas of Anhui Province are approximately 5 years and 11 years, respectively. In recent years, along with the frequent occurrence of extreme hydrological events due to climate change in global or regional scales, a 10-year flood event occurred in 2020 in Anhui Province, which led to about 10.47 million affected people and direct economic losses of CNY 60.07 billion. Meanwhile, Anhui Province also suffered severe autumn drought and continuous drought events, with occurrence frequencies of about 40 and 50 years in 2019 and 2022 separately, which resulted in serious economic losses.

As discussed above, the variability of hydrological variables are the primary disaster-inducing factors of extreme hydrological events; thus, we mainly concentrate on the variability recognition of historical precipitation (P) and temperature (T) sequences in different cities of Anhui Province in this study to explore their impacts on the propagation of the hydrological disaster system. The data series applied in this study mainly includes historical monthly-scale precipitation and temperature indicators, 1960–2020, in different cities of Anhui Province, which can be obtained through the Anhui Provincial Statistical Yearbook, Chinese Meteorological Data Service Centre (http://data.cma.cn, accessed on 10 May 2024) and the Global Land Data Assimilation System (GLDAS) (https://ldas.gsfc.nasa.gov/gldas/, accessed on 10 May 2024).

### 2.2. Research Framework

**Figure 2:**
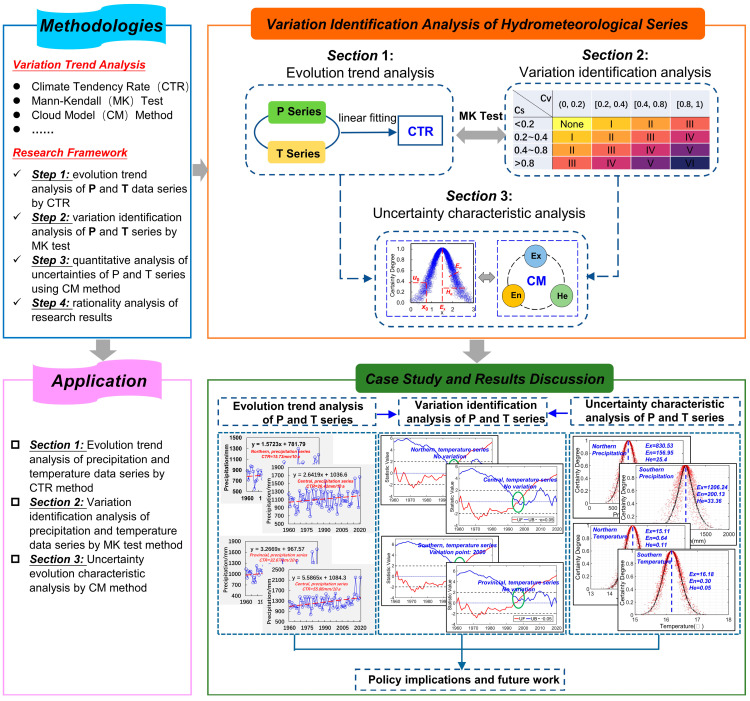
Research framework of variability identification and uncertainty characteristic analysis of historical P and T series in Anhui Province, China.

On the whole, by means of the utilization of the Linear Tendency Rate (LTR), Mann–Kendall (M–K) Test Method, and Cloud Model (CM) approach, we built the research framework of variability identification and uncertainty characteristic analysis of hydrological variables in Anhui Province, China, in this study, which is clearly demonstrated in Figure 2.

As indicated in Figure 2, the primary objective of this study is to recognize the variability points of historical P and T sequences by means of LTR and M–K trend test methods and then quantitatively reveal the uncertainty characteristic using the CM method in Anhui Province, China. Therefore, in Section 1, Methodologies, firstly, we explicitly introduce the fundamental approaches, including the LTR, M–K test, and CM methods. We then show that the theoretical analysis of this study primarily comprises three sections, including the monthly, annual, and interdecadal variability characteristic analysis of historical P and T series through the LTR index; variability point identification by means of the M–K method; and a comparative analysis of the uncertainty features before and after the variability points using CM method. In Section 2, Application Analysis, we discuss in detail the application results of the established research framework in Anhui Province, China, to demonstrate its reliability. In addition, we also explain the application shortcomings, suggestions, and limitations of the proposed variability identification and uncertainty characteristic analysis framework of hydrological variables and the future research directions of this study.

## 3. Methodologies

### 3.1. Linear Tendency Rate (LTR)

In this study, we utilized the Linear Tendency Rate (LTR) method based on one-variable linear regression equation to reveal the historical variation features of P and T sequences. In other words, if parameters *y_i_* and *x_i_* denote a stochastic variable and its corresponding time separately, then the one-variable linear regression equation between the variable *y_i_* and its time series *x_i_* can be represented as follows [15,24,25]:

(1) $$y_{i}=a\cdot x_{i}+b\;\;(i=1\sim n)$$

where parameter *a* is regression coefficient, which represents the variation trend of random variable sequence *y_i_* over time series *x_i_*. If *a* > 0, it indicates that the variable *Y* presents linear increasing trend during the study period, and *a* < 0 indicates that the variable *Y* presents linear decreasing trend. Parameter *b* is constant and can be obtained through least squares method [24]. In this study, we utilize parameter *a* × 10 (unit, °C/10a) to represent the LTR of historical P and T sequences, i.e., LTR = 10*a*.

### 3.2. Mann–Kendall (M–K) Test Method

The M–K method is a non-parametric statistical test method designed to assess the variability trend of non-normality distribution variables, characterized by computational simplicity. In addition, one key advantage of this method is its independence from specific distributional assumptions and robustness against the influence of outliers [26,27]. The M–K test method can not only diagnose the evolution trend features of random variables but is also capable of identify its variability points during historical years. Meanwhile, to further express the significance level of variability trend of variable sequences, the confidence level *α* of M–K trend test was set at 95%, with the corresponding critical values of statistic equaling ±1.96. Therefore, for stochastic variable series *X* = (*x*_1_, *x*_2_, *x*_3_, …, *x_n_*), based on the construction of the sample cumulative count rank sequence {*r_j_*} satisfying *x_i_* > *x_j_* (1 ≤ *j* ≤ *i*), the statistic series {*S_k_*} can be defined as follows:

(2) $$r_{j}=\left\{\begin{array}{ll} 1 & x_{i}>x_{j}\\ 0 & x_{i}\leq x_{j} \end{array}\right.\;\;\;(j=1,\;2,\;\cdots ,\;n)$$

(3) $$S_{k}=\sum_{j=1}^{k}r_{j}\;\;\;(k=1,\;2,\;\cdots ,\;n)$$

Assuming the series {*S_k_*} satisfies independent identically distribution, then the statistics *UB_k_* and *UF_k_* can be constructed [27,28] as follows:

(4) $$UF_{k}=\frac{S_{k}-E\left(S_{k}\right)}{\sqrt{Var\left(S_{k}\right)}}\;\;(k=1,\;2,\;\cdots ,\;n)$$

(5) $$UB_{k}=-UF_{k}$$

(6) $$E\left(S_{k}\right)=\frac{n\left(n-1\right)}{4}$$

(7) $$Var\left(S_{k}\right)=\frac{n\left(n-1\right)\left(2n+5\right)}{72}$$

where *E*(*s_k_*) is the expectation of series {*S_k_*}, and *Var*(*S_k_*) denotes the variance of the stochastic sequence {*S_k_*}. If plotting the statistics *UF_k_* and *UB_k_* in the same coordinate system and letting significance level *α* = 95%, then the two critical lines corresponding to *U*_1−*a*/2_ = ±1.96 can be obtained, and the variability points of sequence *X* can also been identified through the variation trends of statistics *UB_k_* and *UF_k_*, i.e., if there is an intersection between *UB_k_* and *UF_k_* within the critical lines, then it can be indicated that variability point exists for variable sequence *X* (satisfying *α* = 95%), and the intersection point can be identified as the beginning time of variability process of variable *X* [26,27,28].

Generally, the variability points derived by different identification methods are not always the same; thus, we divided variability levels according to the overall changing degrees of average and variability coefficient *C_v_* for the variable sequences before and after the variability points, and proposed the calculation formulas of overall changing degrees $\mu_{\bar{x}}$ and $\mu_{Cv}$ of average and variability coefficient of the variable sequences before and after variability points [14,28] as follows:

(8) $$\mu_{\bar{x}}=\left|({\bar{x}}_{1}-{\bar{x}}_{2})/{\bar{x}}_{2}\right|$$

(9) $$\mu_{C_{v}}=\left|(Cv_{1}-Cv_{2})/Cv_{2}\right|$$

where $\mu_{\bar{x}}$ and $\mu_{Cv}$ denote the overall changing degrees of average and variability coefficient of variable sequence, ${\bar{x}}_{1}$ and ${\bar{x}}_{2}$ represent the average values for the variable sequences before and after the variability points, and $Cv_{1}$ and $Cv_{2}$ represent the variability coefficients for the variable sequences before and after the variability points. Then, the division criterion of variability level based on the overall changing degrees $\mu_{\bar{x}}$ and $\mu_{Cv}$ of stochastic variable sequences can be determined [14,28], which includes six levels, i.e., level 0 (no variability), level 1 (weak variability), level 2 (light variability), level 3 (moderate variability), level 4 (strong variability), level 5 (severe variability), and level 6 (extreme variability), as illustrated in Table 1.

### 3.3. Cloud Model (CM)

Cloud Model (CM) method is an uncertain mathematical cognitive model proposed by Professor D. Y. Li from China to achieve the conversion from a qualitative concepts to its corresponding quantitative characteristic parameters in the 1990s [29,30]. The definition of CM can be represented as follows, letting *U* be a universal set denoted by precise data, and *C* is a qualitative concept within *U*. If plot *x*(*x* ∊ *U*) is a random realization of concept *C* and satisfies normal distributions of *x*~*N*(*Ex*, *En*′^2^) and *En*′~*N*(*En*, *He*^2^), then the membership degree of sample plot *x* belonging to concept *C* can be defined as certainty degree *μ*(*x*) [29] as follows:

(10) $$\mu (x)=\exp\left[-\frac{{\left(x-Ex\right)}^{2}}{2En^{\prime 2}}\right]$$

where data plot (*x*, *μ*(*x*)) is called a cloud drop, and the distribution of concept *C* can be defined as cloud, denoted as *C*(*Ex*, *En*, *He*). In other words, the distribution features of concept *C* can be quantitatively described by three parameters, namely, average *Ex*, entropy *En*, and hyper-entropy *He*. (1) The average *Ex* is the center of sample plot distribution and can best represent the properties of cloud concept ***C*** [31]. (2) Entropy *En* is a measure of uncertainties, including randomness and fuzziness of cloud concept *C*. On the one hand, entropy *En* can reflect the dispersion degree of massive sample plot distribution of concept cloud: the smaller *En*, the smaller dispersion degree, and the more concentrated distribution of sample plots, which is a reflection of randomness. On the other hand, entropy *En* also denotes the membership degree of different cloud plot distributions of concept cloud; the smaller the entropy *En*, the higher the certainty degree of diverse cloud plots belonging to concept cloud, which is reflection of fuzziness. In this study, the smaller the entropy value of cloud distribution, the more concentrated the sample distribution, and the lower the uncertainty levels of the corresponding concept cloud distribution. (3) Hyper-entropy *He* is a measure of the uncertainty of entropy *En*, which was determined by the randomness and fuzziness of entropy *En* and makes cloud drops uniformly distributed on both sides of average *Ex* [29,30]. In addition, the smaller the hyper-entropy *He*, the thinner and more concentrated the distribution map of cloud plots, and the more stable and lower uncertainty the variation of the concept cloud distribution. In this study, the smaller the hyper-entropy *He* of the P and T sample distribution, the more stable and the lower uncertainty the current concept cloud distribution and the lower the likelihood of the occurrence of variability.

**Figure 3:**
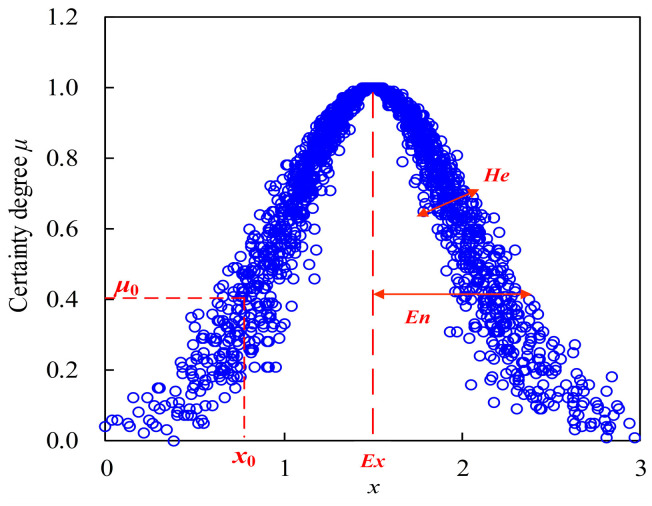
Sample distribution of cloud concept *C*(*Ex* = 1.5, *En* = 0.6, *He* = 0.1).

In this study, the CM approach was innovatively utilized to comparatively discuss the uncertainty features of diverse concept cloud distribution of historical P and T sequences before and after variability points by means of three cloud characteristic parameters *Ex*, *En*, and *He.* Figure 3 demonstrated the sample data distribution of concept cloud *C*(*Ex* = 1.5, *En* = 0.6, *He* = 0.1) as follows:

### 3.4. Calculation Procedures

To sum up, the main intention of this study is to identify the variability points and derive the uncertainty characteristics of P and T sequences by means of the LTR, M–K test, and CM methods. Based on the introduction of the methodology background above, the calculation procedures of this study can be summarized as follows:

Step 1: variation trend and characteristic analysis of historical P and T series using the LTR index. The variation trend and features of P and T series on monthly, annual, and interdecadal scales in northern, central, southern, and provincial areas of Anhui Province were implemented by means of the LTR method in this study.

Step 2: identification of variability points of P and T series using the M–K test method. We recognized the variability points of P and T series using the M–K test method and also determined different variability levels according to the overall derived changing degrees $\mu_{\bar{x}}$ and $\mu_{Cv}$ of average and variability coefficients before and after the variability points (as illustrated in Table 1).

Step 3: uncertainty characteristic analysis of P and T series using the CM method. In this study, based on the obtain of cloud plot distribution map of P and T series before and after the variability points, the Backward Cloud Generator (BCG) method was employed to describe the uncertainty features by means of the three cloud characteristic parameters, i.e., average *Ex*; entropy *En*; hyper-entropy *He*; and the calculation formulas of *Ex*, *En*, and *He* can be denoted as follows [29,30,31]

(11) $$Ex=\frac{1}{n}\sum_{i=1}^{n}x_{i}$$

(12) $$En=\sqrt{\frac{\pi }{2}\times }\frac{1}{n}\sum_{i=1}^{n}\left|x_{i}-Ex\right|$$

(13) $$He=\sqrt{S-En^{2}}$$

where *x_i_* represents different cloud plot of P and T series, and variable *S* denotes the second-order eccentricity of P and T sequences.

## 4. Results and Discussion

### 4.1. Evolution Trend Analysis of Historical Precipitation and Temperature Series

The evolution trend characteristic analysis of P and T sequences is the fundamental issue of variability identification and uncertainty analysis, and the historical variation features of P and T series was discussed on monthly, annual, and interdecadal scales from 1960 to 2020 in Anhui Province in this study.

#### 4.1.1. Monthly Variation Trend Analysis

**Figure 4:**
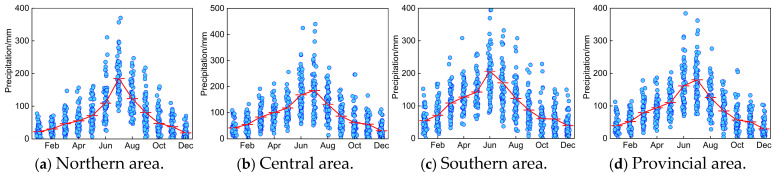
Monthly variation of historical P series in Anhui Province.

**Figure 5:**
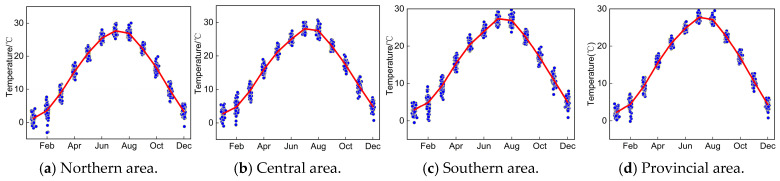
Monthly variation of historical T series in Anhui Province.

Based on the historical data series of precipitation and temperature during 1960–2020, we discussed the monthly variation features of P and T sequences in northern, central, southern, and entire provincial areas, as indicated in Figure 4 and Figure 5.

It can be concluded in Figure 4 and Figure 5 that (1) the monthly distribution difference of annual P series in different areas of Anhui Province was remarkable during 1960 to 2020, which primarily appears during flood season from June to September. In detail, the monthly variation of annual P series is comparatively more obvious in northern and central areas. Anhui Province exhibits distinct climatic zonation, presenting transitional regions between a semi-humid warm temperate monsoon climate in the north and a humid subtropical monsoon climate in the south. This climatic transition creates distinct precipitation gradients, particularly across the Huaihe River Basin. Meanwhile, the elevational difference between the southern mountainous area and northern plain results in an uneven spatial distribution of precipitation. (2) The overall variation in annual P series presents a gradual increasing trend in different areas of Anhui Province, and the maximum and minimum of annual precipitation of entire Anhui Province occurred in 2003 (1288.53 mm) and 1978 (644.95 mm), respectively. Accordingly, extreme severe flood and drought disasters also occurred in 2003 and 1978 throughout the entirety of Anhui Province. In 2003, the Yangtze River Basin experienced a catastrophic flood event due to persistent heavy rainfall and a large influx of water from upstream areas. In contrast, in 1978, due to the impact of a subtropical high-pressure system, this region experienced severe drought. (3) It is clear that the monthly variation differences in temperature in different areas of Anhui Province are remarkable, and a higher temperature primarily occurs from June to September, which is beneficial for the growth of crops.

#### 4.1.2. Annual Variation Features Analysis

**Figure 6:**
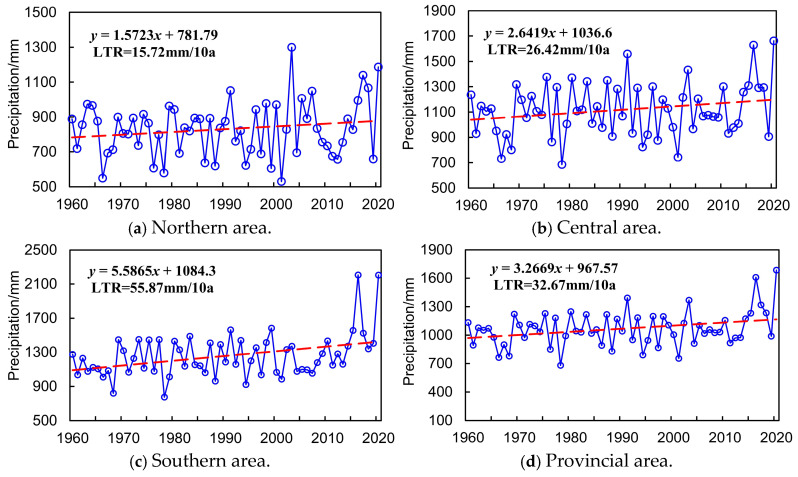
Annual variation of historical P series in Anhui Province.

**Figure 7:**
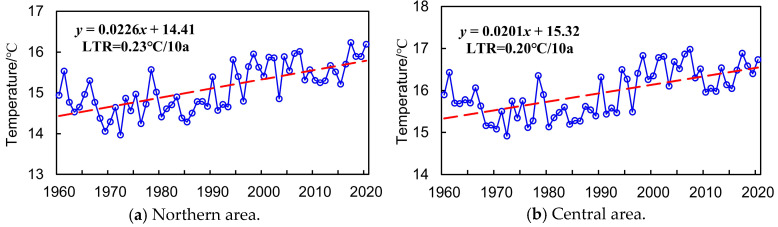

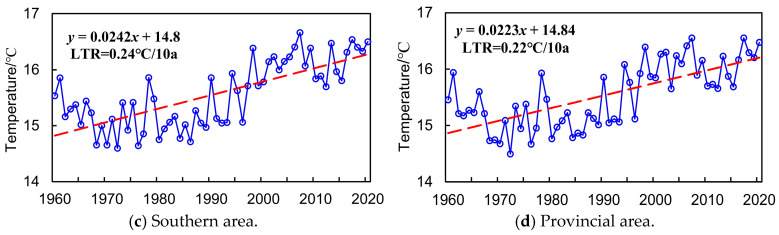
Annual variation of historical T series in Anhui Province.

The LTR index was applied to quantitatively reveal the interannual variation trend and evolution features of P and T series throughout 1960 to 2020 in northern, central, southern, and entire provincial areas, as indicated in Figure 6 and Figure 7.

It is demonstrated in Figure 6 that (1) the annual precipitation in northern, central, southern, and the entirety of Anhui Province all presented a significant increasing trend from 1960 to 2020, as well as a spatially decreasing trend gradually from south to north. In addition, the annually increasing rate of precipitation is the most significant in the southern area, with the LTR index nearly equaling 55.87 mm/10a, which is remarkably higher than that of the central (LTR = 26.42 mm/10a), northern (LTR = 15.72 mm/10a), and entire Anhui Province (LTR = 32.67 mm/10a). Anhui Province is located in the East Asian monsoon region. The summer monsoon introduces a large amount of water vapor from the ocean. The southern area is closer to the source of water vapor and more strongly influenced by the summer monsoon, resulting in more abundant precipitation, which leads to more precipitation in the south and less in the north. (2) The average annual precipitation in northern, central, southern, and the entirety of the province was 830.53 mm, 1118.54 mm, 1257.46 mm, and 1068.84 mm, respectively, with the maximum annual precipitation occurring in 2003 (1300.43 mm), 2020 (1661.57 mm), 2020 (2204.48 mm), and 2020 (1684.25 mm). Specifically, the entire provincial area suffered a serious flood disaster in 2020, with the most extreme rainfall intensity, longest rainy season, largest cumulative rainfall, and widest flood damage area since 1949, and the direct economic loss reached approximately CNY 60.07 billion. (3) The minimum annual precipitation in northern, central, southern, and the entirety of Anhui Province was 530.77 mm (2001), 685.24 mm (1978), 775.26 mm (1978), and 679.85 mm (1978). Severe spring, summer, and autumn continuous drought events occurred in Anhui Province, especially in 1978, with the drought-affected agricultural acreage accounting for 98% of the total, which is the most typical drought disaster with the longest drought period, widest damage area, and most severe disaster loss since 1949.

Meanwhile, it can also be concluded from Figure 7 that (1) the annual average temperature of the northern, central, southern, and entire provincial area presented an obvious increasing trend during 1960–2020, with the LTR index equaling about 0.22 °C/10a in the entirety of Anhui Province, which varies similarly in different areas. (2) The mean annual average temperature in the northern, central, southern, and entire Anhui Province was 15.11 °C, 15.79 °C, 15.55 °C, and 15.53 °C, respectively, in which the maximum annual average temperature was 16.23 °C (2017), 16.97 °C (2007), 16.66 °C (2007), and 16.55 °C (2017). The minimum of annual average temperature was 13.96 °C (1972), 14.91 °C (1972), 14.59 °C (1972), and 14.49 °C (1972), and the spatial variation characteristic of the annual average temperature was not obvious during 1960–2020 in Anhui Province. The north–south latitude span of Anhui Province is relatively small, resulting in limited differences in solar radiation. In addition, the entire province is influenced by a relatively unified monsoon circulation. The strong synchrony of temperature changes further weakens the spatial differences in temperature.

#### 4.1.3. Interdecadal Variation Characteristics Analysis

**Figure 8:**
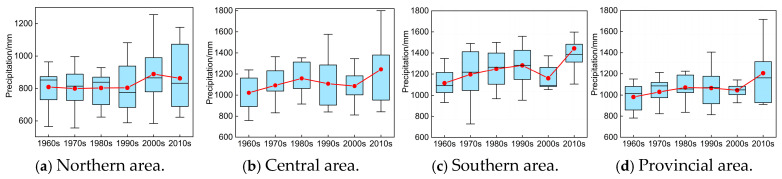
Variation in historical P series during different eras in Anhui Province (note: the red line chart represents the average variation in P sequences).

**Figure 9:**
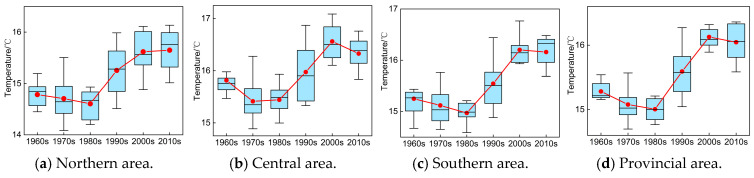
Variation in historical T series during different eras in Anhui Province (note: the red line chart represents the average variation in T sequences).

The box-plot was frequently utilized to reveal the concentration or dispersion distribution situation of sample scatters. Here, we also provide the box-plot of historical P and T sequences from 1960 to 2020 in different areas of Anhui Province to discuss their interdecadal variation characteristics, as illustrated in Figure 8 and Figure 9.

It is demonstrated in Figure 8 that (1) overall, the annual precipitation in different eras of the southern area was much greater than that of the same periods in the central and northern areas, and the increase in annual precipitation in southern area is more evident especially after 2000s than that of the central and northern areas. In addition, except for the 1960s, 2000s, and 2010s, with relatively lower concentration degrees, the annual precipitation in most years of the 1970s, 1980s, and 1990s basically approached the average value of the same periods in the southern area, and the variation range of precipitation series gradually shrunk from 1960 to 2020. (2) The trend of increase in the P series was not obvious, while the corresponding variation range was gradually enlarging in northern and central areas from 1960 to 2020. Meanwhile, in northern and central areas, the annual precipitation in most years before 1990 was basically greater than the average value of the same periods; however, this is exactly the contrary after 1990. (3) Regarding the entire provincial area, the distribution range of historical P series did not vary remarkably from 1960 to 2020, and the concentration degree of P series was basically equivalent to or greater than the average value of the same periods, except for the 1980s and 2010s.

Similarly, it is revealed in Figure 9 that (1) the overall increasing trend of historical T sequences was consistent in different areas of Anhui Province, which presented a slight decreasing trend from 1960 to 1990 but increased significantly after 1990. (2) The variation range of T series became larger after 1990, especially in the northern and central areas, and the maximum variation range appeared in the 1990s in different areas. (3) The overall concentration degree of T series was approximately equivalent to or lower than the average value of the same periods in different areas of Anhui Province, except for the 2010s.

### 4.2. Variability Recognition Analysis According to M–K Trend Test Method

**Figure 10:**
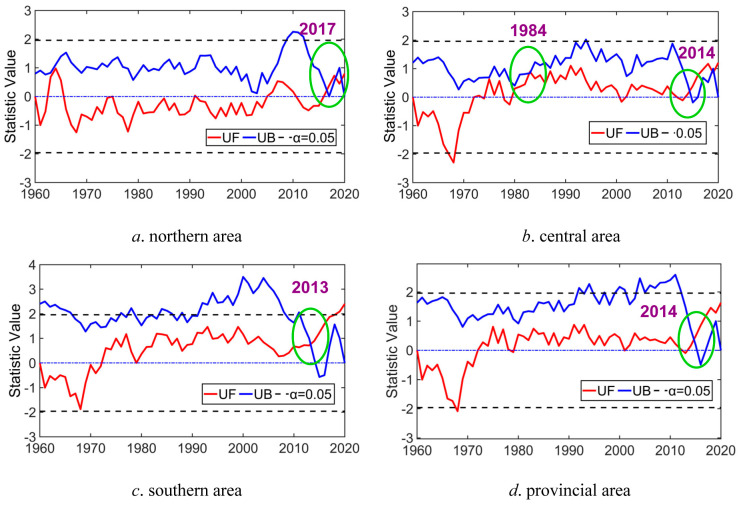
Variability trend recognition of historical P series in Anhui Province (note: the green circle denotes the variability point).

**Figure 11:**
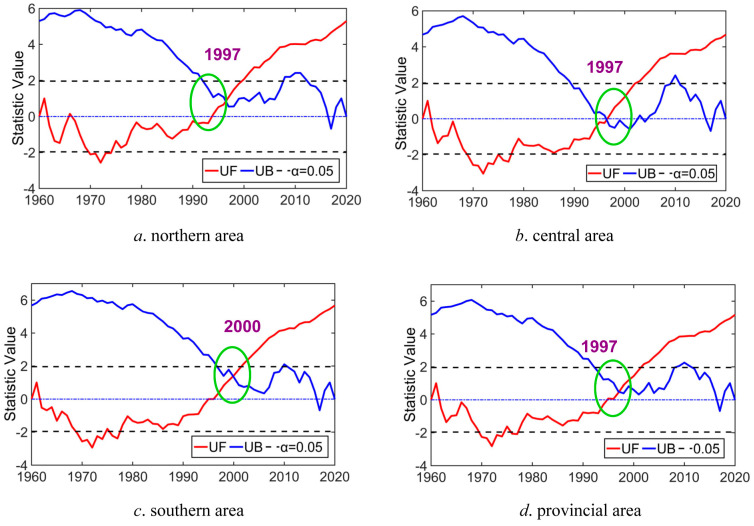
Variability trend recognition of historical T series in Anhui Province (note: the green circle denotes the variability point).

Based on the variation feature discussion of P and T series in Anhui Province, we also utilized the M–K test method to identify the variability points of historical P and T series in 1960–2020, and the corresponding identification results of the variability points of P and T series in different areas are displayed in Figure 10 and Figure 11.

It can be concluded by looking at Figure 10 and Figure 11 that (1) the variation curves of statistical parameters *UB* and *UF* of the P sequence in North Anhui Province during 1960–2020 intersect at 2017 and 2018, and the intersection point varies within the threshold level of significance level *α* equaling 95%, indicating that significant changes or variability of the P series appeared in 2017 in the northern area. Similarly, evident change or variability of the historical P sequence also occurred in 1983 and 2014 in the central area, in 2013 in the southern area, and in the entire provincial area in 2014. In other words, significant change or variability exists for the P series in the entirety of Anhui Province in 1960–2020. (2) Likewise, the change curves of *UB* and *UF* of the T series in the northern area in 1960–2020 intersect at 1997, and the intersection point varies within the threshold curves of *α* = 95%; thus, it can be concluded that obvious changes or variability in the T series occurred in 1997 in the northern area. Evident change or variability of T sequence also occurred in 1997 in the central area, in 2000 in the southern area, and in 1997 in the entire provincial area. That is to say, obvious changes or variability of temperature series also occurred in the entirety of Anhui Province in 1960–2020.

Using the identified variability points of historical P and T sequences in different areas of Anhui Province for demarcation, the entirety of historical P and T sequences can be divided into two parts, corresponding to before and after the variability points. Then, the variability grades of historical P and T sequences in different areas of Anhui Province can be determined by means of the division criterion of the variability levels of the stochastic variable based on $\mu_{\bar{x}}$ and $\mu_{Cv}$ (as indicated in Table 1). The diagnosis result is displayed in Table 2 and Table 3.

On the whole, it is revealed in Table 2 and Table 3 that light variability (level 2) of precipitation in southern and entire provincial areas and temperature in the southern area occurred in 1960–2020, and the overall variability levels of P and T series in different regions of Anhui Province were not significant from 1960 to 2020 (under level 2, light variability).

### 4.3. Uncertainty Characteristic Analysis by CM Method

**Figure 12:**
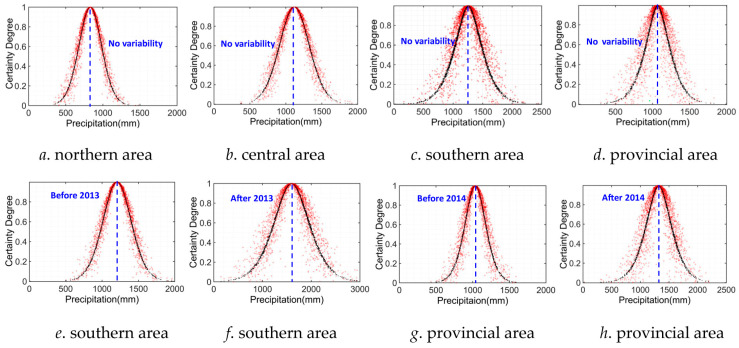
Cloud distribution of P data series in Anhui Province.

In this section, based on the identification results of variability points of historical P and T series in different areas of Anhui Province, we further derived the cloud characteristic parameters, including average *Ex*, entropy *En*, and hyper-entropy *He* of diverse cloud distributions corresponding to original data sequences or separated data series of P and T indicators using variability points over level 2 (light variability). The cloud characteristic parameters of *Ex*, *En*, and *He* were determined by means of the BCG algorithm to comparatively discuss the uncertainty characteristic of historical precipitation variation in different areas, as indicated in Table 4, and the corresponding cloud distribution is also displayed in Figure 12.

It can be concluded by looking at Table 4 and Figure 12 that (1) According to the average value *Ex* of different cloud distributions of P sequences, on the whole, the annual precipitation is the greatest in the southern area but lowest in the northern area regardless of whether considering variability influences or not from 1960 to 2020, and the annual precipitation increased significantly after the variability points 2013 and 2014 separately in the southern and entire provincial areas. For instance, the average annual precipitation increased from 1206.24 mm to 1596.79 mm in the southern area and from 1036.26 mm to 1320.19 mm in the entirety of Anhui Province. Especially, the annual precipitation of southern and entire provincial areas reached 2204.48 mm and 1684.25 mm in 2020, respectively, which led to the average annual precipitation increasing rapidly after variability points. (2) Based on the entropy value *En* of different cloud distributions of P sequences, the entropy value *En* is the greatest (243.36) in the southern area and lowest (156.95) in the northern area without considering its variability features, which indicates that the varying randomness of the P series was the lowest in the northern area, i.e., the certainty degree was the highest in the northern area, and this is exactly the contrary in the southern area. In addition, the entropy value *En* of the P series increased from 200.13 to 380.89 in the southern area and from 148.58 to 233.86 in the entire provincial area before and after the variability points 2013 and 2014, respectively, which reveals that the random uncertainty characteristic of P series were aggravated remarkably after variability points 2013 and 2014 in the southern and entire provincial areas. (3) It can be also inferred from hyper-entropy *He* that the hyper-entropy value *He* is the greatest (86.32) in the southern area and lowest (25.42) in the northern area if ignoring its variability features, which also reveals that the varying fuzziness of P series was the lowest in the northern area and highest in the southern area, i.e., the distribution thickness of cloud plots is thinner in the northern area (as shown in Figure 12a), and the varying certainty degree of the P series was the highest in the northern area as well. Moreover, the hyper-entropy value *He* of the P series increased from 33.36 to 101.00 in the southern area and from 34.62 to 74.93 in the entire provincial area before and after variability points 2013 and 2014 separately, which also indicates that the fuzzy uncertainty degrees of the P series were intensified after variability points 2013 and 2014 in the southern and provincial areas.

**Figure 13:**
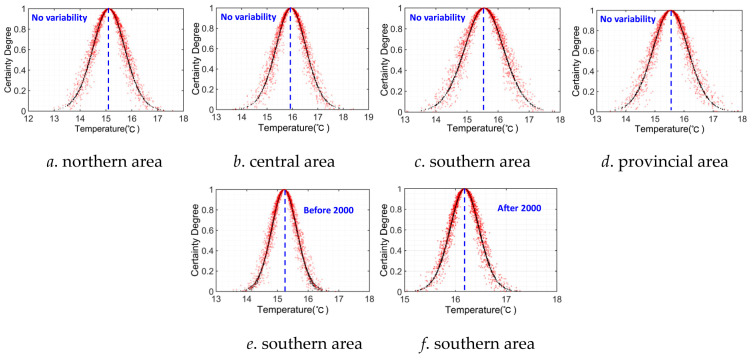
Cloud distribution of historical T series in Anhui Province.

Likewise, we also derived the cloud characteristic parameters of *Ex*, *En*, and *He* of different cloud distributions of the T series using the BCG algorithm to explore its uncertainty characteristics in different areas, which is given in Table 5, and the corresponding cloud distribution is also provided in Figure 13.

It can be concluded from Table 5 and Figure 13 that (1) according to the cloud characteristic value of the average *Ex* of T sequences, the changing historical T series is not clear if ignoring its variability features in different areas of Anhui Province. The annual average temperature only increased from 15.22 °C to 16.18 °C before and after variability point 2000 in the southern area. (2) According to the entropy value *En* of the different cloud distributions of T sequences, the entropy value *En* is the greatest (0.65) in the southern area and lowest (0.61) in the central area without consideration for its variability features, which indicates that the random uncertainty degree was the highest in the southern area and lowest in the central area. However, this is totally different when considering the variability features of T series, i.e., the random uncertainty degree of the T series reduced in the southern area when considering its variability features, which is the lowest in the entire provincial area, while this is exactly the contrary in the northern area. (3) It can also be inferred from hyper-entropy *He* that the difference in hyper-entropy value *He* among different areas is subtle if ignoring its variability features. However, the hyper-entropy value *He* of T series became lower before and after variability point 2020 in the southern area. In other words, the fuzzy uncertainty degree of T series was remarkably alleviated in the southern area when considering its variability features. Accordingly, the cloud distribution thickness of sample plots became thinner in the southern area (as shown in Figure 13e), i.e., the varying certainty degree of the T series was the highest in the southern area as well.

All in all, based on the evolution characteristic analysis and variability identification of P and T series in different areas of Anhui Province, we comparatively and quantitatively discussed the uncertainty characteristics of historical P and T sequences by means of three cloud characteristic parameters, *Ex*, *En*, and *He*. On the whole, the uncertainty features of P series were evidently intensified after variability points 2013 and 2014 in the southern and provincial areas, respectively, but were remarkably alleviated for T series when considering its variability features in 2020 in the southern area. The obtained research results will be of great significance for scientifically explaining the spatio-temporal evolution trend and characteristics of hydrological sequences.

## 5. Conclusions

In this study, in the context of impacts of climate change and human activities based on the evolution characteristic analysis and variability identification of annual P and T sequences in Anhui Province, we explored the uncertainty features of historical P and T series using three cloud characteristic parameters, average *Ex*, entropy *En*, and hyper-entropy *He* of the CM approach. This is also the main intention and novelty of this study. Eventually, we summarized the primary findings, shortcomings of this study, and provided the future research directions as follows:

(1) Overall, the annual precipitation in Anhui Province presented a significant increasing trend from 1960 to 2020 and a decreasing trend from south to north. Specifically, the increasing rate of precipitation is the most significant in the southern area, with LTR = 56.87 mm/10a. The maximum annual precipitation of the central, southern, and entire provincial areas appeared in 2020. In addition, the minimum annual precipitation of the central, southern, and entire Anhui Province all occurred in 1978. Moreover, the annual average temperature of the entire provincial area presented an obvious increasing trend in 1960–2020, with the LTR equaling about 0.22 °C/10a.

(2) Significant variability of the P series appeared in 2017 in the northern area (level 1, weak variability), in 2013 in the southern area (level 2, light variability), and in 2014 in the entire provincial area (level 2, light variability). Likewise, obvious variability of the T series occurred in 1997 in the northern area (level 1, weak variability) and in 2000 in the southern area (level 2, light variability).

(3) Entropy *En* and hyper-entropy *He* of the historical P series all increased obviously before and after the variability points in the southern and entire provincial areas, which revealed that the uncertainty features of P series were evidently intensified after 2013 and 2014 in the southern and provincial areas, respectively. However, the entropy *En* and hyper-entropy *He* of historical T series all became much lower before and after its variability point 2020 in the southern area, indicating that the fuzzy uncertainty of the T series was remarkably alleviated in the southern area when considering its variability features.

In conclusion, the evolution characteristic analyses and variability identification of hydrological variables is a fundamental issue to establish reasonable hydrological models, and the variation recognition results of hydrological indicators can be applied to modify the initial parameter calibration results of a prediction model for future scenarios, which will be beneficial to improve prediction accuracy. In addition, due to the evolution of hydrological variables presenting inconsistency and even mutation features, the original design standard of water conservancy projects (reservoirs, dams, etc.) based on the consistency hypothesis of historical hydrological data needs an upgrade to improve its operation security. Thus, the integration of research results obtained in this study will be of great benefit for users. In addition, further discussions focusing on the variability identification method, derivation of cloud characteristic parameters under diverse variability scenarios, and rationality analysis of results will also be conducted. Many related studies in terms of the driving factors of recognition resulting in variability features as well as dynamic adjustment of hydrological model parameters considering variability characteristics still need to be conducted, which is a future direction of this study.

## Figures and Tables

**Table 1:** Division criterion of variability levels of variable based on $\mu_{\bar{x}}$ and $\mu_{Cv}$.

$\mu_{\bar{x}}$	Variability Level			
	$\mu_{Cv}$ < 0.2	$0.2\;<\;\mu_{Cv}$ ≤ 0.4	$0.4\;<\;\mu_{Cv}$ ≤ 0.8	$\mu_{Cv}$ > 0.8
$\mu_{\bar{x}}$ < 0.2	Level 0 (no variability)	Level 1 (weak)	Level 2 (light)	Level 3 (moderate)
0.2 < $\mu_{\bar{x}}$ ≤ 0.4	Level 1 (weak)	Level 2 (light)	Level 3 (moderate)	Level 4 (strong)
0.4 < $\mu_{\bar{x}}$ ≤ 0.8	Level 2 (light)	Level 3 (moderate)	Level 4 (strong)	Level 5 (severe)
$\mu_{\bar{x}}$ > 0.8	Level 3 (moderate)	Level 4 (strong)	Level 5 (severe)	Level 6 (extreme)

**Table 2:** Variability identification result of P series in Anhui Province.

Area	Variability Point	$\mu_{\bar{x}}$	$\mu_{Cv}$	Variability Level
Northern area	2017	0.1954	0.2587	level 1 (weak variability)
Central area	1983	0.0654	0.0787	level 0 (no variability)
	2014	0.1876	0.1693	level 0 (no variability)
Southern area	2013	0.2492	0.3787	level 2 (light variability)
Provincial area	2014	0.2237	0.2176	level 2 (light variability)

**Table 3:** Variability identification result of T series in Anhui Province.

Area	Variability Point	$\mu_{\bar{x}}$	$\mu_{Cv}$	Variability Level
Northern area	1997	0.0571	0.2934	level 1 (weak variability)
Central area	1997	0.0502	0.1789	level 0 (no variability)
Southern area	2000	0.0595	0.5852	level 2 (light variability)
Provincial area	1997	0.0570	0.1913	level 0 (no variability)

**Table 4:** Cloud characteristic values of P series in Anhui Province.

Area	Variability Point	*Ex*	*En*	*He*
Northern area	No variability	830.53	156.95	25.42
Central area	No variability	1118.54	212.22	35.37
Southern area	Before variability point (2013)	1206.24	200.13	33.36
	After variability point (2013)	1596.79	380.89	101.34
	No variability	1257.50	243.36	86.62
Provincial area	Before variability point (2014)	1036.26	148.58	34.62
	After variability point (2014)	1320.19	233.86	74.93
	No variability	1068.82	174.57	66.64

**Table 5:** Cloud characteristic values of T series in Anhui Province.

Area	Variability Point	*Ex*	*En*	*He*
Northern area	No variability	15.11	0.64	0.11
Central area	No variability	15.94	0.61	0.10
Southern area	Before variability point (2000)	15.22	0.42	0.07
	After variability point (2000)	16.18	0.30	0.05
	No variability	15.55	0.65	0.11
Provincial area	No variability	15.53	0.63	0.10

## Data Availability

The original contributions presented in this study are included in the article. Further inquiries can be directed to the corresponding author.
